# Neoagarohexaose Attenuates Inflammatory and Oxidative Joint Injury in MIA/CIOA Mouse Models of Osteoarthritis

**DOI:** 10.3390/ijms27146162

**Published:** 2026-07-10

**Authors:** Nan Wu, Yating Du, Chaocheng Wu, Zhuhua Chan, Runying Zeng

**Affiliations:** Technology Innovation Center for Exploitation of Marine Biological Resources, Third Institute of Oceanography, Ministry of Natural Resources, Xiamen 361005, China; wunan@tio.org.cn (N.W.);

**Keywords:** neoagarohexaose, osteoarthritis, inflammation, oxidative stress, topical delivery

## Abstract

Osteoarthritis (OA) is a prevalent chronic joint disease lacking disease-modifying drugs. Animal models with distinct pathogenic mechanisms—monosodium iodoacetate (MIA) for metabolic toxicity and collagenase-induced osteoarthritis (CIOA) for matrix degradation—are essential for therapeutic evaluation. In this study, topical application of neoagarohexaose (NA6) at 5 and 10 mg/kg twice daily was assessed in MIA- and CIOA-induced mouse OA models. NA6 at both doses reduced paw swelling, improved serum oxidative stress markers (catalase (CAT), malondialdehyde (MDA), myeloperoxidase (MPO)), ameliorated cartilage damage and Osteoarthritis Research Society International (OARSI) scores, and decreased inflammatory cell infiltration. Immunohistochemistry showed that NA6 downregulated interleukin-1β (IL-1β) and interleukin-6 (IL-6), upregulated NAD(P)H:quinone oxidoreductase 1 (NQO1), and restored the compensatorily elevated heme oxygenase-1 (HO-1) toward baseline levels. The high-dose NA6 (10 mg/kg) showed comparable or favorable efficacy at the tested doses relative to the positive control diclofenac. These results demonstrate that NA6 exerts anti-inflammatory, antioxidant, and chondroprotective effects in both OA models, supporting its potential as a topical therapeutic candidate for OA symptom management and structural protection.

## 1. Introduction

Osteoarthritis (OA) is a chronic degenerative joint disease characterized by progressive articular cartilage degeneration, subchondral bone remodeling, and synovial inflammation. As one of the most common joint disorders, OA imposes a heavy burden on global public health systems [1]. However, no disease-modifying drug capable of effectively slowing or reversing OA progression has been approved to date. Current treatment options remain largely limited to symptomatic management with non-steroidal anti-inflammatory drugs (NSAIDs), which temporarily relieve pain and inflammation but fail to halt ongoing cartilage degeneration [2]. Therefore, the discovery and development of novel therapeutic agents with anti-inflammatory, antioxidant, and chondroprotective effects has become a major focus of OA research.

The pathogenesis of OA is complex and involves multiple interacting factors, among which inflammation and oxidative stress play central roles in disease onset and progression [3,4]. Under pathological conditions, locally overexpressed inflammatory cytokines such as interleukin-1β (IL-1β) and interleukin-6 (IL-6) activate various signaling pathways, inducing chondrocyte apoptosis and extracellular matrix degradation [5,6]. Concurrently, oxidative stress resulting from excessive reactive oxygen species (ROS) accumulation further exacerbates articular cartilage damage and is a major contributor to early chondrocyte death [7,8]. To counteract oxidative stress, the body initiates endogenous antioxidant defense mechanisms, among which the nuclear factor erythroid 2-related factor 2 (Nrf2) signaling pathway serves as a key antioxidant response hub [8]. Upon activation, Nrf2 upregulates the expression of various downstream antioxidant enzymes, including heme oxygenase-1 (HO-1) and NAD(P)H quinone oxidoreductase-1 (NQO1), thereby scavenging excess ROS and maintaining intracellular redox homeostasis. Previous studies have shown that NQO1 expression is downregulated while HO-1 is compensatorily elevated in many OA models, suggesting impaired local antioxidant defense [8]. Targeting the Nrf2/HO-1 signaling pathway to enhance endogenous antioxidant capacity is considered a potential therapeutic strategy for OA.

Neoagarohexaose (NA6) is a novel marine-derived functional oligosaccharide obtained from the hydrolysis of red algal agarose, exhibiting good water solubility and bioavailability [9,10]. Accumulating evidence indicates that NA6 and related neoagarooligosaccharides (NAOs) possess various biological activities, including antioxidant and anti-inflammatory effects [10,11]. In vitro studies have demonstrated that NAOs, with NA6 as a representative active monomer, effectively scavenge DPPH radicals and inhibit intracellular ROS production [12]. Recent studies have further shown that NA6 and related NAOs can activate the Nrf2/HO-1 antioxidant pathway and suppress the release of pro-inflammatory cytokines such as IL-1β and IL-6 in activated macrophages [13]. However, the in vivo anti-inflammatory and antioxidant protective effects of NA6 specifically in animal models of osteoarthritis have not yet been systematically reported. The specific effects of NA6 on alleviating articular cartilage injury, ameliorating oxidative stress imbalance, and regulating NQO1 and HO-1 expression remain to be elucidated.

Topical administration represents an advantageous route for local OA treatment, as it avoids first-pass metabolism, increases local drug concentration in the joint, and reduces systemic adverse effects, making it particularly suitable for chronic disease management requiring long-term medication. Based on these considerations, the present study adopted topical application of NA6 in two mouse OA models induced by monosodium iodoacetate (MIA) and collagenase (CIOA), respectively. We comprehensively evaluated the effects of NA6 on paw swelling, histopathological changes, proteoglycan loss, and OARSI scores in OA animals. Furthermore, by detecting the expression levels of IL-1β, IL-6, NQO1, and HO-1 in joint tissues, we preliminarily explored the anti-inflammatory and antioxidant molecular mechanisms of NA6, aiming to provide experimental evidence for the application of marine-derived oligosaccharides in the topical treatment of osteoarthritis.

## 2. Results

### 2.1. NA6 Alleviates MIA-Induced Paw Swelling and Oxidative Stress in Mice

**Figure 1:**
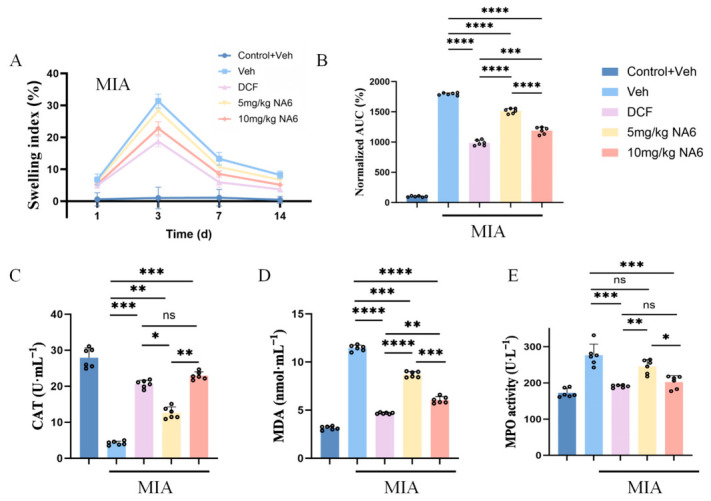
NA6 alleviates MIA-induced paw swelling (reflecting ankle joint edema) and improves oxidative stress markers in mice. (**A**) Time-course of paw swelling index in each group after MIA injection. (**B**) Area under the curve (AUC) of paw swelling index. (**C**–**E**) Serum biochemical parameters on day 14: (**C**) CAT activity, (**D**) MDA content, (**E**) MPO activity. Data are mean ± SD. Statistical analysis was performed using one-way ANOVA with Tukey’s post hoc test. * *p* < 0.05, ** *p* < 0.01, *** *p* < 0.001, **** *p* < 0.0001; ns, not significant. N = 6.

Paw swelling was monitored on days 1, 3, 7, and 14 after MIA modeling (Figure 1A). The control group showed little change in paw thickness, remaining near baseline throughout the experiment. In contrast, MIA model mice developed rapid and pronounced paw swelling, peaking at day 3 (swelling rate ~31.37%), followed by a gradual decline. Compared with the model group, all treatment groups alleviated paw swelling to varying degrees. The positive control diclofenac (DCF) group exhibited the most significant inhibition (peak ~18.7%), followed by the NA6 high- and low-dose groups. At days 3 and 7, the NA6 high-dose group showed statistically significant differences compared with the model group. Area under the curve (AUC) analysis (Figure 1B) further confirmed that all treatment groups had significantly lower overall swelling levels compared with the model group, indicating an anti-inflammatory effect of NA6.

Oxidative stress markers (Figure 1C–E) showed that MIA modeling significantly disrupted redox balance, as evidenced by decreased CAT activity and increased MDA and MPO levels. After drug intervention, these abnormalities were ameliorated to varying extents. The NA6 high-dose group showed a more pronounced effect in restoring CAT activity, outperforming the DCF group, whereas DCF was more effective in reducing MDA and MPO levels. These results indicate that NA6 ameliorates systemic oxidative stress imbalance induced by MIA, as evidenced by the restoration of serum antioxidant capacity and reduction in lipid peroxidation markers.

### 2.2. NA6 Alleviates CIOA-Induced Paw Swelling and Oxidative Stress in Mice

**Figure 2:**
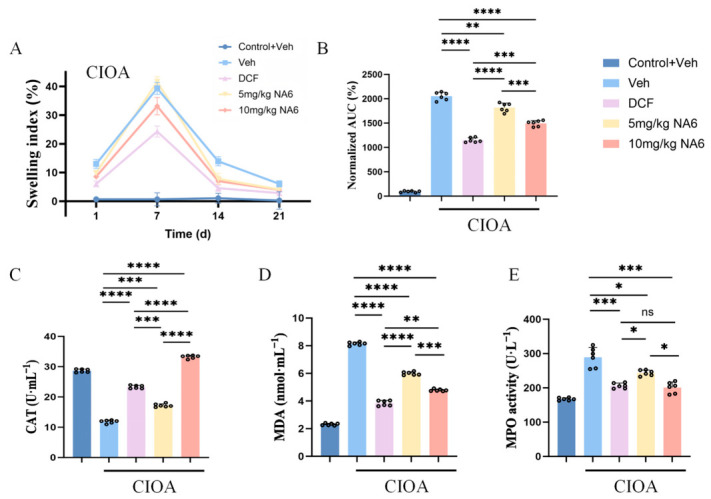
NA6 ameliorates CIOA-induced paw swelling (reflecting ankle joint edema) and oxidative stress in mice. (**A**) Time-course of paw swelling index in each group after CIOA injection. (**B**) AUC of paw swelling index. (**C**–**E**) Serum biochemical parameters on day 21: (**C**) CAT activity, (**D**) MDA content, (**E**) MPO activity. Data are mean ± SD. Statistical analysis was performed using one-way ANOVA with Tukey’s post hoc test. * *p* < 0.05, ** *p* < 0.01, *** *p* < 0.001, **** *p* < 0.0001; ns, not significant. N = 6.

Paw swelling and oxidative stress markers were also evaluated in the CIOA-induced mouse model. As shown in Figure 2A, CIOA modeling induced obvious paw swelling, which increased and then gradually declined. Compared with the model group, all treatment groups reduced paw swelling to varying degrees, with the DCF group showing the most pronounced inhibition, followed by the NA6 high-dose group, while the low-dose group showed relatively limited improvement. At days 7 and 14, the NA6 high-dose group showed statistically significant differences compared with the model group. AUC analysis (Figure 2B) was consistent with this trend.

Oxidative stress markers (Figure 2C–E) showed that CIOA modeling similarly decreased antioxidant capacity and increased lipid peroxidation, as indicated by reduced CAT activity and elevated MDA and MPO levels. After NA6 intervention, these abnormalities were ameliorated to varying extents. Similarly to the findings in the MIA model, the NA6 high-dose group showed favorable regulatory effects in restoring CAT activity and reducing MDA levels, while its effect on reducing MPO levels was slightly inferior to that of DCF. Collectively, NA6 exhibited significant anti-swelling effects and systemic antioxidant modulation in the CIOA model, with trends generally consistent with those observed in the MIA model.

### 2.3. NA6 Improves Joint Histological Damage and Reduces Inflammatory Cell Infiltration in Both MIA and CIOA Models

**Figure 3:**
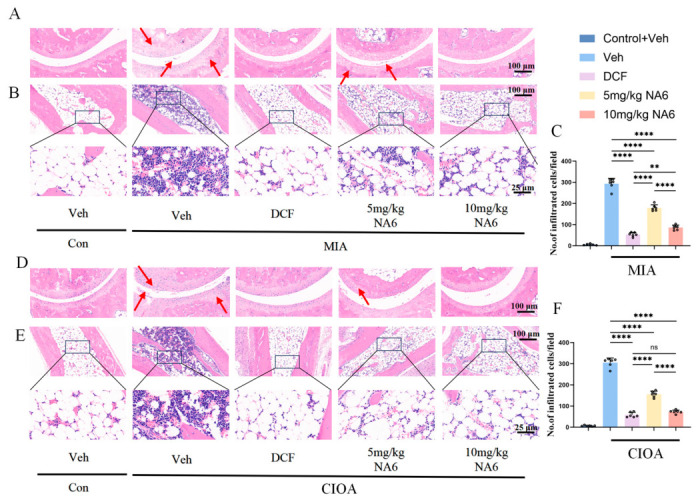
NA6 improves ankle joint histopathological damage and reduces inflammatory cell infiltration in MIA and CIOA mice. (**A**,**D**) HE staining showing articular cartilage structure in MIA (**A**) and CIOA (**D**) mice (scale bar: 100 μm). Red arrows indicate irregular or damaged cartilage surfaces. (**B**,**E**) HE staining and magnified views of inflammatory cell infiltration in the bone marrow region (scale bars: 100 μm and 25 μm). (**C**,**F**) Quantification of infiltrated inflammatory cells per field. Data are mean ± SD. Statistical analysis was performed using one-way ANOVA with Tukey’s post hoc test. ** *p* < 0.01, **** *p* < 0.0001; ns, not significant. N = 6.

Histological analyses were performed on dissected ankle joints. HE staining revealed obvious histological damage in the ankle joints of both MIA and CIOA model mice (Figure 3A,D), characterized by an uneven, rough, or defective articular cartilage surface, disrupted cartilage layer structure, and disorganized or morphologically abnormal chondrocytes, indicating pronounced degenerative changes. After NA6 intervention, these histological damages were ameliorated to different extents: the cartilage surface became smoother and more continuous, the structural integrity of the cartilage layer was partially restored, and chondrocyte arrangement became progressively organized. The high-dose NA6 group exhibited more pronounced improvement. These changes were consistent between the MIA and CIOA models.

Examination of local joint inflammation (Figure 3B,E) showed extensive infiltration of inflammatory cells, mainly neutrophils and monocytes/macrophages, in the ankle joint tissues of the model groups, indicating a marked enhancement of local inflammatory responses. NA6 treatment significantly reduced the degree of inflammatory cell infiltration and decreased the number of inflammatory cells. Inflammatory cell counts (Figure 3C,F) were consistent with the histological observations: the model groups had significantly higher numbers of inflammatory cells compared with the control groups, while both the NA6 high-dose group and the DCF group significantly reduced inflammatory cell counts. Collectively, NA6 effectively alleviated articular cartilage structural damage and inhibited inflammatory cell infiltration in both models, indicating stable protective effects against cartilage injury and local inflammation.

### 2.4. NA6 Ameliorates Cartilage Proteoglycan Loss and Reduces OARSI Scores

**Figure 4:**
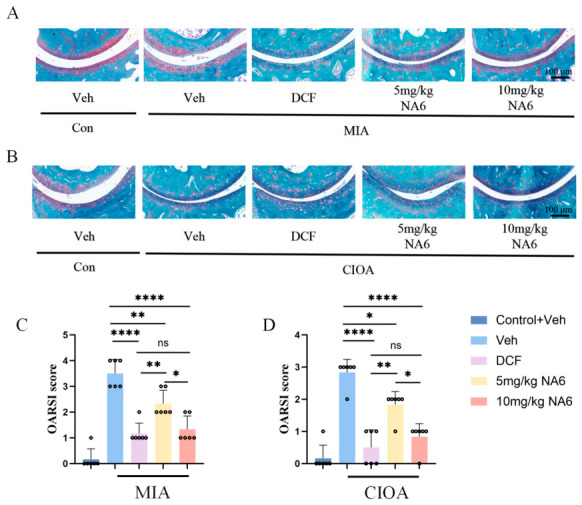
NA6 protects articular cartilage and reduces OARSI pathological scores. (**A**,**B**) Safranin O-Fast Green staining showing proteoglycan distribution and structural integrity of ankle joint cartilage in MIA (**A**) and CIOA (**B**) mice (scale bar: 100 μm). (**C**,**D**) Pathological scores based on the OARSI scoring system. Data are mean ± SD. Statistical analysis was performed using one-way ANOVA with Tukey’s post hoc test. * *p* < 0.05, ** *p* < 0.01, **** *p* < 0.0001; ns, not significant. N = 6.

Safranin O-Fast Green staining was performed on ankle joint sections, and cartilage damage was quantitatively assessed using the OARSI scoring system (Figure 4A–D). In both the MIA and CIOA models, marked cartilage degeneration was observed after modeling, characterized by significantly diminished or locally absent Safranin O staining, indicating substantial proteoglycan loss, accompanied by an irregular cartilage surface and disrupted matrix structure. NA6 intervention alleviated cartilage degeneration to varying degrees, as evidenced by increased Safranin O staining intensity, restoration of proteoglycan content, and gradual recovery of matrix structure. The high-dose NA6 group exhibited more pronounced improvement.

OARSI scores (Figure 4C,D) further supported the histological observations. Compared with the model groups, all NA6 treatment groups had significantly lower scores, and the high-dose NA6 group achieved scores lower than those of the DCF group, approaching the levels of the control group. In the MIA model, the NA6 high-dose group showed a statistically significant difference compared with the DCF group. Taken together, NA6 reduced cartilage proteoglycan loss, improved matrix structural integrity, and lowered cartilage damage scores in both models, suggesting stable protective effects against cartilage degeneration.

### 2.5. NA6 Downregulates IL-1β and IL-6 Expression in Joint Tissues

**Figure 5:**
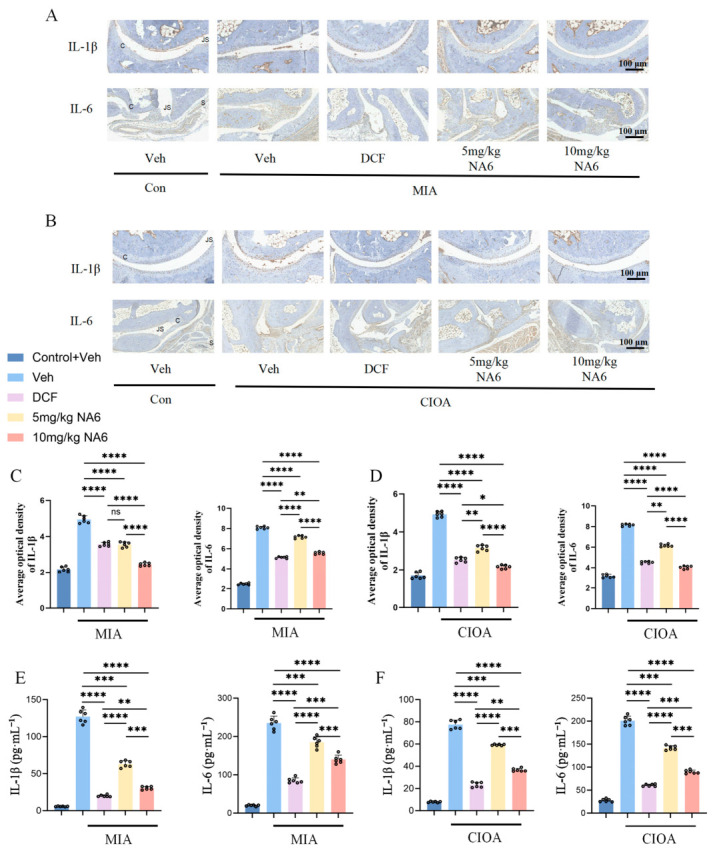
NA6 inhibits IL-1β and IL-6 expression in joint tissues and serum of MIA and CIOA mice. (**A**,**B**) Representative IHC staining of IL-1β and IL-6 in ankle joints of MIA (**A**) and CIOA (**B**) mice. All images were captured at ×400 magnification from the mid-zone of the tibial plateau cartilage. Scale bar: 100 μm. Abbreviations: C, cartilage; JS, joint space; S, synovium. (**C**,**D**) Quantitative analysis of average optical density (IOD/area) of IL-1β and IL-6 in joint tissues of MIA (**C**) and CIOA (**D**) models. (**E**,**F**) Serum concentrations of IL-1β and IL-6 measured by ELISA in MIA (**E**) and CIOA (**F**) models. Data are presented as mean ± SD. Statistical analysis was performed using one-way ANOVA with Tukey’s post hoc test. * *p* < 0.05, ** *p* < 0.01, *** *p* < 0.001, **** *p* < 0.0001; ns, not significant. N = 6.

The expression levels of IL-1β and IL-6 in ankle joint tissues were detected by immunohistochemistry (Figure 5A,B). In both MIA and CIOA models, positive expression of IL-1β and IL-6 was markedly enhanced after modeling, as evidenced by expanded positive staining areas and increased staining intensity, indicating significant local joint inflammation. After NA6 intervention, these inflammatory cytokines were markedly reduced in a dose-dependent manner, with the high-dose (10 mg/kg) group showing the most pronounced suppression. Quantitative immunohistochemistry (IHC) analysis confirmed that all treatment groups significantly lowered the average optical density of IL-1β and IL-6 compared with the model groups (Figure 5C,D). Notably, the NA6 high-dose group exhibited a trend toward greater suppression of IL-1β relative to the positive control DCF, while its effect on IL-6 downregulation was comparable to that of DCF. To further confirm the systemic anti-inflammatory effects, serum levels of IL-1β and IL-6 were measured by ELISA (Figure 5E,F). The results showed that NA6 significantly reduced the serum concentrations of both cytokines in a dose-dependent manner, closely paralleling the decreased IHC expression in joint tissues. Taken together, these findings demonstrate that NA6 effectively suppresses IL-1β and IL-6 expression at both the local joint and systemic levels, thereby alleviating the inflammatory response in OA.

### 2.6. NA6 Modulates NQO1 and HO-1 Expression in Joint Tissues

**Figure 6:**
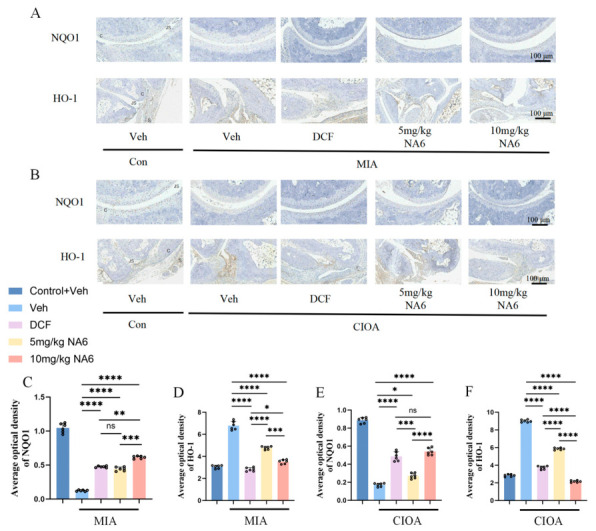
NA6 modulates NQO1 and HO-1 expression in joint tissues of MIA and CIOA mice. (**A**,**B**) Representative immunohistochemical staining of NQO1 and HO-1 in ankle joints of MIA (**A**) and CIOA (**B**) mice. All images were captured at ×400 magnification from the mid-zone of the tibial plateau cartilage. Scale bar: 100 μm. Abbreviations: C, cartilage; JS, joint space; S, synovium. (**C**–**F**) Quantitative analysis of average optical density (IOD/area): (**C**) NQO1 in the MIA model; (**D**) HO-1 in the MIA model; (**E**) NQO1 in the CIOA model; (**F**) HO-1 in the CIOA model. Data are presented as mean ± SD. Statistical analysis was performed using one-way ANOVA with Tukey’s post hoc test. * *p* < 0.05, ** *p* < 0.01, *** *p* < 0.001, **** *p* < 0.0001; ns, not significant. N = 6.

The expression levels of NQO1 and HO-1 in ankle joint tissues were detected by immunohistochemistry (Figure 6). In both the MIA and CIOA models, NQO1 positive expression was significantly decreased after modeling, while HO-1 expression was markedly increased, indicating altered local antioxidant enzyme homeostasis. After NA6 intervention, NQO1 expression was significantly upregulated, as shown by expanded positive staining areas and increased staining intensity; meanwhile, the elevated HO-1 expression was restored toward normal levels, as evidenced by narrowed positive staining areas and decreased staining intensity. The high-dose NA6 group exhibited the most pronounced regulatory effects. Quantitative analysis was consistent with the histological observations, showing that all treatment groups had significantly higher NQO1 expression levels, while HO-1 expression was restored toward that of the control group.

## 3. Discussion

Two mechanistically complementary mouse models—MIA (metabolic toxicity-driven) and CIOA (matrix degradation/mechanical instability-driven)—were used to evaluate topical NA6 in OA. NA6 significantly reduced paw swelling, improved histopathological damage, lowered OARSI scores, and restored proteoglycan content. In both models, NA6 downregulated IL-1β and IL-6, upregulated NQO1, and restored the compensatorily elevated HO-1 toward baseline. The high-dose NA6 group showed comparable or favorable outcomes relative to DCF at the tested doses in several measures. These results indicate that NA6 exerts both anti-inflammatory and antioxidant effects, and shows protective actions on cartilage in two distinct OA models.

The MIA and CIOA models represent two distinct mechanisms of OA pathogenesis. MIA induces chondrocyte metabolic toxicity and rapid necrosis by inhibiting GAPDH, with intense inflammation appearing before structural damage [14]. CIOA degrades intra-articular collagens, causing joint instability and secondary cartilage degeneration, with inflammation developing later [15,16]. Despite these differences in initial injury, disease progression, and the timing of inflammation, NA6 regulated IL-1β, IL-6, NQO1, and HO-1 similarly in both models. This consistency suggests that NA6 does not target specific upstream triggers but acts on a common downstream pathway linking inflammation and oxidative stress [17]. The MIA and CIOA models were chosen for their complementary pathogenic drivers and experimental convenience; nevertheless, we recognize that surgical instability models are required to assess efficacy in mechanically induced OA, and such studies are planned.

NA6 at 5 and 10 mg/kg reduced IL-1β and IL-6 protein expression in joint tissues, and serum ELISA further confirmed that NA6 significantly reduced circulating IL-1β and IL-6 levels (Figure 5E,F), consistent with the local tissue findings. These changes were associated with reduced paw swelling, less inflammatory cell infiltration, and lower OARSI scores. In the MIA model, the high-dose NA6 group showed a trend toward greater suppression of IL-1β than DCF (1 mg/kg). In the CIOA model, NA6 and DCF showed comparable effects. One possible explanation for this difference is that the two models involve different inflammatory processes. MIA induces a rapid and intense inflammatory response, while CIOA has a slower, more gradual course (21 days vs. 14 days) and may engage a broader range of inflammatory signals [18]. In this more complex setting, the relative advantage of NA6 over DCF became less pronounced. Importantly, NA6 reduced both IL-1β and IL-6 simultaneously. Blocking these two key cytokines together may be more effective at dampening the inflammatory response than targeting a single pathway [19]. DCF was used as the positive control in both models, which is consistent with clinical guidelines for topical OA therapy [20]. However, DCF primarily exerts anti-inflammatory effects and does not directly address the oxidative stress prominent in MIA or the matrix degradation in CIOA. This mechanistic mismatch may partly explain why NA6 high-dose significantly outperformed DCF in OARSI scores in the MIA model, where its antioxidant capacity could confer additional protection against metabolic toxicity, whereas in CIOA the advantage was less pronounced without a structure-protective comparator. Future studies incorporating antioxidant or matrix-protective positive controls would help clarify these model-specific contributions.

Immunohistochemical analysis revealed that in both models, NQO1 expression decreased after model induction, while HO-1 increased, suggesting dysregulation of Nrf2-associated antioxidant enzymes in OA joints [21]. After NA6 treatment, NQO1 was upregulated and the elevated HO-1 was restored toward baseline levels. This pattern indicates that NA6 does not indiscriminately activate all genes downstream of the Nrf2 pathway but rather rebalances antioxidant enzyme homeostasis by alleviating the pathological stimulus that drives compensatory HO-1 overexpression. Sustained high levels of HO-1 have been linked to free iron accumulation and oxidative damage [22]; by attenuating the underlying oxidative stress, NA6 allowed HO-1 to return toward baseline, which may be beneficial for long-term use in chronic diseases.

In parallel, serum biochemical assays demonstrated that NA6 improved systemic CAT activity (the high-dose group performed better than DCF) and lowered circulating MDA and MPO levels. It is important to distinguish that serum markers reflect peripheral/systemic redox status and do not directly quantify joint-localized oxidative stress. Nevertheless, the concordant improvement in both systemic serum parameters and local joint tissue enzyme expression (NQO1 and HO-1) collectively supports that NA6 exerts antioxidant modulation at multiple levels. However, while IHC analysis of NQO1 and HO-1 indicates modulation of local antioxidant enzyme expression, it does not constitute a direct measurement of ROS production or oxidative damage in articular cartilage. Direct approaches such as dihydroethidium (DHE) fluorescent staining for superoxide detection, or biochemical assays for oxidative stress markers in joint homogenates (e.g., TBARS, GSH/GSSG ratio), would provide more definitive evidence for local joint protection. Whether the observed chondroprotection is primarily driven by local joint antioxidant effects, systemic redox improvement, or a combination of both remains to be clarified by future studies incorporating these direct joint tissue-specific measurements alongside pharmacokinetic profiling.

The anti-inflammatory and antioxidant effects are related. Fewer inflammatory cytokines lead to less ROS production, and reduced oxidative stress can limit the amplification of inflammatory signals [23]. NA6 affects both aspects, which may help explain why it achieved a statistically significant reduction in OARSI scores compared with DCF in the MIA model, whereas in the CIOA model a similar trend was observed without significant between-group difference. As DCF acts mainly on inflammation, the added antioxidant capacity of NA6 may confer additional chondroprotection. OA cartilage damage involves both inflammation and oxidative stress, so targeting both may be more effective than targeting inflammation alone.

Compared with other marine-derived substances, fucoidan has a high molecular weight and low oral bioavailability, whereas low-molecular-weight fucoidan derivatives are required to improve absorption [24]. Chitosan has poor solubility at neutral pH, limiting its direct therapeutic application without nanoparticle modification [25]. Glucosamine sulfate shows weak anti-inflammatory activity. NA6 has a favorable balance in molecular weight (~0.99 kDa), water solubility, skin permeability, and stability. In addition, agarose degradation products are generally recognized as safe (GRAS) by the FDA [10]. Traditional cartilage protective agents such as chondroitin sulfate and glucosamine mainly act by supplementing the matrix or inhibiting degrading enzymes, with weak and slow direct effects on inflammatory cytokines. Curcumin and resveratrol have NF-κB inhibitory activity but poor stability and low bioavailability. In both OA models, NA6 showed anti-inflammatory effects comparable to or better than DCF, indicating clear competitive advantages.

NA6 was applied to the skin in this study. With a molecular weight of approximately 0.99 kDa and favorable water solubility, NA6 possesses theoretically suitable physicochemical properties for transdermal formulation development. However, direct evidence that NA6 crosses the skin barrier and reaches the joint at therapeutic concentrations is currently lacking; pharmacokinetic studies measuring percutaneous absorption and joint tissue distribution are warranted. Compared with oral DCF, topical administration avoids first-pass metabolism and gastrointestinal irritation, which is helpful for long-term treatment [20]. Compared with intra-articular injection, skin application is non-invasive and easier for patients to follow. Topical NSAIDs such as diclofenac are recommended as first-line therapy for OA in current guidelines, with systemic uptake limited to approximately 5% [20].

Several limitations should be acknowledged. First, Nrf2 involvement was inferred from downstream targets NQO1 and HO-1, but nuclear translocation was not directly measured; future confirmation requires Nrf2 inhibitors or knockout models. Second, Western blotting on joint tissues was not performed due to limited sample availability; future studies with larger animals or tissue pooling are needed. Third, upstream (e.g., TLR4/NF-κB) and downstream (MMPs, ADAMTS) pathways remain unexplored. Fourth, the chemical models used do not fully reflect chronic mechanical OA; destabilization of the medial meniscus (DMM) models would strengthen translational relevance. Fifth, direct local ROS or oxidative damage measurements were not performed and warrant future investigation. Finally, pain behavior and transdermal pharmacokinetics were not evaluated; although the molecular weight and water solubility of NA6 suggest skin penetration potential, direct evidence of joint distribution after topical application is lacking. Future studies with validated LC-MS/MS methods are needed to confirm percutaneous absorption and guide formulation development. These limitations will be addressed in follow-up experiments.

## 4. Materials and Methods

### 4.1. Experimental Animals

Male C57BL/6J mice aged 6–8 weeks were purchased from Fuzhou Wushi Experimental Animal Trading Co., Ltd. (Fuzhou, China). Mice were housed in the Experimental Animal Center of the Technology Innovation Center for Marine Biological Resources Development under specific pathogen-free conditions, with free access to food and water, five mice per cage. All animal experiments were approved by the Institutional Animal Care and Use Committee of the Third Institute of Oceanography (Approval No. TIO-IACUC-03-2025-07-09).

### 4.2. Drugs and Reagents

Neoagarohexaose (NA6, purity 92.34%) was prepared in our laboratory as previously described [26]. The purity and structural identity of the prepared NA6 were verified by high-performance liquid chromatography (HPLC) and electrospray ionization mass spectrometry (ESI-MS). HPLC analysis was performed on a C18 column (4.6 × 250 mm, 5 μm) with an evaporative light scattering detector (ELSD) or UV detector, using water and acetonitrile as the mobile phase. The retention times of the NA6 standard and the prepared NA6 sample were observed at 20.48 min and 20.42 min, respectively. ESI-MS analysis (operating in both positive and negative ion modes) confirmed the molecular weight, with observed major signals at *m*/*z* 959.27 [M + Na]^+^ and *m*/*z* 935.29 [M − H]^−^, which are consistent with the theoretical molecular weight of NA6. The purity of the NA6 batch was determined to be 92.34% based on the peak area normalization method of the HPLC chromatogram. The representative HPLC and MS chromatograms are shown in Supplementary Figure S1.

Diclofenac sodium (DCF, positive control) was purchased from Thermo Fisher Scientific (Waltham, MA, USA). Monosodium iodoacetate (MIA) and collagenase (CIOA) were obtained from Merck Millipore (Burlington, MA, USA). Kits for catalase (CAT), malondialdehyde (MDA), and myeloperoxidase (MPO) were purchased from Solarbio (Beijing, China). Antibodies against IL-1β, IL-6, NQO1, and HO-1 were obtained from Aifang Biotechnology (Changsha, China). Hematoxylin–eosin (HE) staining kit and Safranin O-Fast Green cartilage staining kit were purchased from Solarbio (Beijing, China). Other reagents were of analytical grade. NA6 and DCF were dissolved in sterile PBS freshly before use. The doses of NA6 (5 and 10 mg/kg) and DCF (1 mg/kg) were selected based on preliminary experiments and literature reports [27,28].

### 4.3. Establishment of Osteoarthritis Models and Drug Administration

#### 4.3.1. MIA-Induced Osteoarthritis Model

The MIA-induced OA model was established according to the method of Zhizheng Fang et al. [27]. MIA solution (0.75 mg/10 μL) was prepared in saline, stirred in the dark, and filter-sterilized through a 0.22 μm membrane. Mice were anesthetized with 2.5% isoflurane inhalation, and a single intra-articular injection of 10 μL MIA solution was administered into the right ankle joint. Control mice received an equal volume of sterile saline. Successful model establishment was determined by the appearance of obvious joint swelling and lameness of the affected limb within 3–7 days after injection. After successful modeling, mice were randomly divided into the following five groups (n = 6 per group): control group (Control + Veh, right ankle injected with saline, treated with topical PBS), model group (MIA + Veh, treated with topical PBS), positive control group (MIA + DCF, treated with topical DCF 1 mg/kg), NA6 low-dose group (MIA + NA6-L, treated with topical NA6 5 mg/kg), and NA6 high-dose group (MIA + NA6-H, treated with topical NA6 10 mg/kg). A 14-day endpoint was selected for the MIA model, as this time frame consistently captures the peak of inflammation and established cartilage lesions, while avoiding the spontaneous partial recovery and increased variability reported in longer durations. All test agents were administered by topical skin application starting on the day after modeling. The fur on the right hind paw (ankle joint area) was gently shaved using an electric clipper 24 h before the first administration and was re-shaved every 3 days thereafter to maintain a hair-free area. 50 μL of drug solution was evenly applied to the skin of the swollen right hind paw (covering the ankle joint area), followed by gentle massage for 30 s to promote absorption. During the procedure, animals were gently restrained by scruffing by a trained technician, allowing full access to the hind paw while keeping the animal immobile. The entire restraint and massage procedure lasted approximately 30–40 s per mouse and was well-tolerated by all animals. Administration was performed twice daily (morning and evening) at fixed times for 14 consecutive days. The control and model groups received an equal volume of PBS.

#### 4.3.2. CIOA-Induced Osteoarthritis Model

The CIOA-induced OA model was established according to the method of Mausset-Bonnefont et al. [29]. Collagenase was dissolved in sterile saline to a working concentration of 2 U/10 μL, filter-sterilized, and injected intra-articularly (10 μL) on day 0 and day 2. Control mice received an equal volume of sterile saline. Successful model establishment was determined by the appearance of obvious swelling within 7–10 days after injection. After successful modeling, mice were randomly divided into the following five groups (n = 6 per group): control group (Control + Veh), model group (CIOA + Veh), positive control group (CIOA + DCF, treated with topical DCF 1 mg/kg), NA6 low-dose group (CIOA + NA6-L, treated with topical NA6 5 mg/kg), and NA6 high-dose group (CIOA + NA6-H, treated with topical NA6 10 mg/kg). A 21-day endpoint was selected for the CIOA model, as this longer time frame is required to allow progressive cartilage degeneration to develop following collagen-induced joint instability, consistent with previous reports. The administration method (topical application twice daily, 50 μL per application, followed by 30 s gentle massage with gentle scruffing restraint) and the shaving procedure for the application site were the same as described in Section 4.3.1. Administration was performed for 21 consecutive days. The control and model groups received an equal volume of PBS.

### 4.4. Measurement of Paw Swelling

On days 1, 3, 7, and 14 (for the MIA model) or days 1, 3, 7, 14, and 21 (for the CIOA model) after modeling, the thickness of the right hind paw (vertical distance from the highest point of the arch to the plantar surface) was measured using an electronic caliper [30]. The measurement was taken at the ankle joint level (the narrowest region proximal to the paw, corresponding to the tibiotarsal articulation), as an indicator of joint swelling [30]. Each mouse was measured three times, and the average value was calculated. The paw swelling rate was calculated as: swelling rate (%) = (post-administration paw thickness − pre-modeling paw thickness)/pre-modeling paw thickness × 100%. The area under the curve (AUC) was also calculated to evaluate the overall swelling level.

### 4.5. Tissue Sample Collection

Twenty-four hours after the last administration, all mice were anesthetized with an intraperitoneal injection of pentobarbital sodium (50 mg/kg), blood was collected from the orbit, and the animals were euthanized. The right hind ankle joints were completely isolated, excess muscle tissue was removed, and the joints were fixed in 4% paraformaldehyde for 24 h, followed by decalcification in 10% EDTA (pH 7.4) for 4 weeks. After gradient ethanol dehydration, the tissues were embedded in paraffin. Serial sections (thickness 5 μm) were prepared for subsequent histological and immunohistochemical staining.

### 4.6. Detection of Oxidative Stress Markers

Blood samples were centrifuged at 3000 rpm for 15 min at 4 °C to separate serum. Serum levels of catalase (CAT) activity, malondialdehyde (MDA) content, and myeloperoxidase (MPO) activity were measured according to the manufacturers’ instructions.

### 4.7. Joint Histopathological Staining

#### 4.7.1. Hematoxylin–Eosin (HE) Staining

Paraffin sections were deparaffinized and rehydrated, stained with hematoxylin for 5 min, blued in tap water, stained with eosin for 3 min, dehydrated through graded ethanol, cleared in xylene, and mounted with neutral resin. Articular cartilage structure, chondrocyte morphology, and inflammatory cell infiltration were observed under a light microscope. For each section, three random high-power fields (×400) were selected, and the number of inflammatory cells (neutrophils and monocytes/macrophages) was counted and averaged.

#### 4.7.2. Safranin O-Fast Green Staining

Paraffin sections were deparaffinized, rehydrated, stained with fast green for 5 min, differentiated with 1% acetic acid, stained with Safranin O for 5 min, dehydrated, cleared, and mounted. The distribution of proteoglycans in the cartilage matrix was observed, with Safranin O-positive areas appearing red. Cartilage damage was quantitatively assessed using the Osteoarthritis Research Society International (OARSI) scoring system: 0 = normal; 1 = mild superficial degeneration; 2 = mild matrix loss; 3 = moderate degeneration or cracks in the middle zone; 4 = deep zone degeneration or severe matrix loss; 5 = full-thickness cartilage degeneration with bone surface exposure [31]. Each section was scored independently by two observers blinded to the group assignments, and the average score was calculated.

### 4.8. Immunohistochemistry

Paraffin sections were deparaffinized and rehydrated. Endogenous peroxidase activity was blocked with 3% H_2_O_2_ for 10 min. Antigen retrieval was performed by microwave heating in sodium citrate buffer (pH 6.0). Non-specific binding was blocked with 10% normal goat serum for 30 min at room temperature. Sections were then incubated with primary antibodies (rabbit anti-mouse IL-1β, 1:200; IL-6, 1:200; NQO1, 1:150; HO-1, 1:150) overnight at 4 °C. After washing with PBS, sections were incubated with HRP-labeled goat anti-rabbit secondary antibody (1:500) for 30 min at room temperature. Signals were visualized with DAB, and sections were counterstained with hematoxylin. The extent and intensity of positive staining (brown granules) were observed under a light microscope. For each section, three random high-power fields (×400) were captured from the mid-zone of the tibial plateau cartilage, ensuring consistent anatomical localization across all samples. Within each captured field, the entire image area was analyzed. The integrated optical density (IOD) and the total area of the field were measured using ImageJ software (version 1.53, National Institutes of Health, Bethesda, MD, USA), and the average optical density was calculated as IOD/area. The mean value from the three fields per section was used for further statistical analysis. The average optical density was measured by two independent observers blinded to group allocation, and the mean value was used for further analysis.

### 4.9. Serum ELISA for IL-1β and IL-6

Serum levels of interleukin-1β (IL-1β) and interleukin-6 (IL-6) were measured using commercial enzyme-linked immunosorbent assay (ELISA) kits (R&D Systems, Minneapolis, MN, USA) according to the manufacturer’s instructions. Briefly, serum samples were diluted 1:2 with assay diluent and added to pre-coated 96-well plates, followed by incubation with detection antibodies and streptavidin-HRP. After washing, substrate solution was added, and the reaction was stopped with stop solution. The absorbance was read at 450 nm using a microplate reader (BioTek, Winooski, VT, USA). Cytokine concentrations were calculated from standard curves generated with known concentrations of recombinant IL-1β and IL-6. All samples were measured in duplicate, and the mean values were used for statistical analysis.

### 4.10. Statistical Analysis

All data are presented as mean ± standard deviation (SD). Normality of distribution was assessed using the Shapiro–Wilk test, and homogeneity of variances was verified using Levene’s test for each dataset. As all data met these assumptions (*p* > 0.05), multiple group comparisons were conducted using one-way analysis of variance (ANOVA), followed by Tukey’s post hoc test for pairwise comparisons. A *p*-value < 0.05 was considered statistically significant. Statistical analysis was performed using GraphPad Prism 8.0 software.

## 5. Conclusions

In summary, topical NA6 in MIA and CIOA mouse models downregulated IL-1β and IL-6, upregulated NQO1, and restored HO-1 to near-normal levels, indicating combined anti-inflammatory and antioxidant actions along with cartilage protection. NA6 worked similarly in two models with different underlying mechanisms, suggesting its effects are not model-specific. In the MIA model, NA6 reduced OARSI scores to a greater extent than DCF. With its moderate molecular weight, good water solubility, favorable physicochemical properties for topical delivery, and a generally recognized safety profile, NA6 may be a candidate for further development as a topical treatment for osteoarthritis.

## Data Availability

Data are available on request from the corresponding author.

## Supplementary Materials

The following supporting information can be downloaded at: https://www.mdpi.com/article/10.3390/ijms27146162/s1.

## Abbreviations

The following abbreviations are used in this manuscript:

NA6	Neoagarohexaose
OA	Osteoarthritis
MIA	Monosodium Iodoacetate
CIOA	Collagenase-Induced Osteoarthritis
DCF	Diclofenac
CAT	Catalase
MDA	Malondialdehyde
MPO	Myeloperoxidase
IL-1β	Interleukin-1 beta
IL-6	Interleukin-6
NQO1	NAD(P)H Quinone Dehydrogenase 1
HO-1	Heme Oxygenase-1
DMM	Destabilization of the Medial Meniscus
ROS	Reactive Oxygen Species
ELISA	Enzyme-Linked Immunosorbent Assay
NSAIDs	Non-Steroidal Anti-Inflammatory Drugs
Nrf2	Nuclear Factor Erythroid 2-Related Factor 2
NF-κB	Nuclear Factor Kappa-B
IHC	Immunohistochemistry
