# Supplementary material for: Neoagarohexaose Attenuates Inflammatory and Oxidative Joint Injury in MIA/CIOA Mouse Models of Osteoarthritis
Source: Int J Mol Sci. 2026 Jul 10;27(14):6162. doi: 10.3390/ijms27146162 (PMC13410720; doi:10.3390/ijms27146162)
Supplement: Supplementary file 1 [file ijms-27-06162-s001.zip › ijms-4394389-supplementary.pdf]

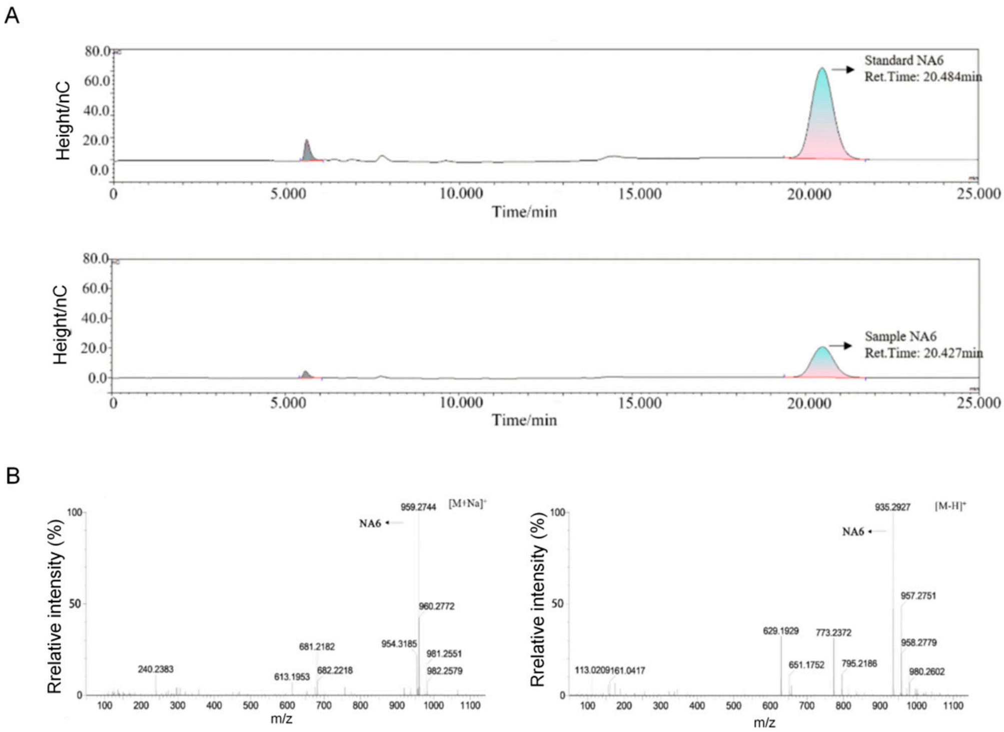


Supplementary Figure S1. Purity analysis of the prepared NA6 sample. (A) HPLC chromatograms of the standard NA6 (upper panel) and the prepared NA6 sample (lower panel). The retention times of the standard and sample were observed at 20.48 min and 20.42 min, respectively. (B) ESI-MS spectra of the NA6 sample in positive ion mode (left, showing the major $[M+Na]^+$ ion at m/z 959.27) and negative ion mode (right, showing the major $[M-H]^-$ ion at m/z 935.29). The purity of the prepared NA6 batch was determined to be 92.34% based on the peak area normalization method of the HPLC chromatogram.
